# Evaluation of MALAT1 promoter DNA methylation patterns in early colorectal lesions and tumors 

**Published:** 2019

**Authors:** Vahid Chaleshi, Shiva Iran, Masoud Alebouyeh, Reza Mirfakhraie, Hamid Asadzadeh Aghdaei

**Affiliations:** 1 *Department of Biology, Science and Research Branch, Islamic Azad University, Tehran, Iran*; 2 *Foodborne and Waterborne Diseases Research Center, Research Institute for Gastroenterology and Liver Diseases, Shahid Beheshti University of Medical Sciences, Tehran, Iran*; 3 *Department of Medical Genetics, Shahid Beheshti University of Medical Sciences, Tehran, Iran*; 4 *Basic and Molecular Epidemiology of Gastrointestinal Disorders Research Center, Research Institute for Gastroenterology and Liver Diseases, Shahid Beheshti University of Medical Sciences, Tehran, Iran *

**Keywords:** Long non-coding RNAs, Colorectal cancer, Colonic polyps, Metastasis-associated lung adenocarcinoma transcript 1, MALAT1, DNA Methylation

## Abstract

**Aim:**

This study set out to determine the effect of methylation on MALAT1 gene in primary colorectal lesions and tumors to gain further knowledge about the diagnostic and prognostic value of MALAT.

**Background:**

Metastasis-associated lung adenocarcinoma transcript 1 (MALAT1) is one of the long non-coding RNAs that plays an important role in invasion, cell proliferation, and metastasis of various cancers. However, there is insufficient information on the association between MALAT1 and the methylation process as well as its role in the development of colorectal cancer.

**Methods:**

Methylation pattern of MALAT1 promoter was determined by Methylation-Specific Polymerase Chain Reaction (MSP) in 86 colorectal primary lesions, tumors, and normal specimens. MALAT1 methylation pattern was compared in tumor and polyp tissue. In order to obtain more accurate results, we investigated the association between MALAT1 promoter methylation pattern and clinicopathologic factors in patients.

**Results:**

The results indicated that the MALAT1 promoter methylation pattern in the tumor tissue, primary lesion tissue, and normal was not significantly different (p=0.430). Comparison of the MALAT1 promoter methylation pattern between polyp types and tumor tissue groups was not significant either (p=0.437). Surprisingly, the methylation frequency of MALAT1 methylation was significantly higher in colon lesions than in their rectum lesion (p = 0.035). In addition, no significant hypermethylation of MALAT1 was observed between the other patients’ clinicopathological data in both polyp 46/66 and tumor tissues 20/66.

**Conclusion:**

This study dealt with determining the effect of methylation on MALAT1 gene in primary colorectal lesions and tumors to gain further knowledge about the diagnostic and prognostic value of MALAT.

## Introduction

 Colorectal cancer is one of the major causes of mortality worldwide and claims over 9% of all prevalence causes of cancer (1, 2). It is the third common type of cancer worldwide and fourth frequent cause of death (3).

Symptoms of the disease include a rapid change in bowel habits, including diarrhea or constipation, rectal or stool bleeding, persistent abdominal discomfort, seizures, or pain (4). These symptoms occur at an early stage of the disease and no diagnosis test can be made for initial stages. Thus, it is most often diagnosed in stages three and four, when cancer spreads throughout the entire body (5). Detection of diagnostic biomarkers can be used to improve the treatment.

Practically, all CRCs progress from colorectal polyps and it can may take years for to become cancerous (6, 7). Polyps have various types, including adenomatous polyp, hyperplastic polyp (hp), inflammatory polyp, etc. It is generally accepted that most adenomatous polyps can be converted to colon carcinoma (8, 9). The prevalence of HP is not clear yet; HPs are the smallest type of polyp and have a very low risk of developing cancer (10). Inflammatory polyps occur more frequently in inflammatory bowel disease (IBD) patients. They are also known as pseudo-polyps, but are produced as a response to chronic inflammation in the colon and are at high risk to develop into colon cancer (11).

Methylation of CpG islands within the promoter region appear to decrease gene expression, though it is only typical of two kinds of methylation that are related with cancer development: type A (for age-related) methylation, and type C (for cancer-specific) methylation (12). 

Noncoding RNAs (ncRNAs) are functional RNA molecules that play an important role in regulating post-transcriptional gene expression. Most ncRNAs interact with RNA-binding proteins (13, 14). However, their mechanism has not yet been fully elucidated (15). LncRNAs play an important role in the pathogenesis of various diseases (16).

Long non-coding RNAs (lncRNAs) are a large and diverse class of transcribed RNA molecules of more than 200 nucleotides in length. Recently, lncRNAs have received much attention in processes such as cancer, metastasis, and drug resistance (17, 18)

Further, LncRNAs appear to have a unique functional potential due to their ability in interacting with almost all biomolecules. Metastasis-associated lung adenocarcinoma transcript 1 (*MALAT1*) is an oncogene associated with tumor invasion in non-small cell lung cancer which is regulated by DNA methylation (19). It is generally overexpressed in the tumor and metastatic state of the disease. Also, a functional study has shown the role of metastatic suppressor for *MALAT1* (20). Overexpression of *MALAT1* has been observed in a large number of tumor types including breast, pancreas, colon, prostate, and liver (17, 21-23). 

*MALAT1* regulates the methylation status of histone chromatin through binding to the target PRC2 complex (24, 25). Due to its multiple roles in different types of cancer, *MALAT1* has recently received much attention as a therapeutic target for clinical applications.

The present study was designed to determine the effect of promoter methylation of *MALAT1* gene in early colorectal lesions and tumors to gain further knowledge about the diagnostic and prognostic value of *MALAT1*. 

## Methods

**Patients and Sampling**

Tissue samples were collected from 66 patients and 20 normal. A total of 20 patients were all first diagnosed with a tumor while 46 patients with colorectal hyperplastic, adenomas, and inflammatory polyp tissues, respectively. Patient consent was received during May 2014 to 2016 from random patient samples referring to the Research Institute for Gastroenterology and Liver Diseases (RIGLD), Taleghani Hospital, Shahid Beheshti University of Medical Sciences, Tehran, Iran, who had undergone a colonoscopic procedure for either screening or polypectomy purposes. Each tissue specimen was separated into two parts. One part of the specimens was fresh frozen in liquid nitrogen and stored at -80 ◦C for further evaluation, while the second part was fixed in cold 10% neutral buffered formalin for 24 h and then embedded in a paraffin block. Histopathological examination of tissue specimens was confirmed following evaluation by a pathologist. Non-Iranian patients and those who had no pathological data were dismissed according to study exclusion criteria. Demographic and clinicopathological data were obtained from the questionnaire and histopathology reports. Location of tumor was divided into colon and rectum. Cancers were staged according to the Tumor-NodeMetastasis (TNM) classification of the Union for International Cancer Control (UICC) (26). 

**Genomic DNA Extraction and Sodium Bisulfite Treatment**

DNA was extracted from a freshly frozen tissue using Qiagen QIAamp DNA Mini Kit (Cat. 51324, Qiagen, Germany) according to the manufacturer’s instructions (QIAamp DNA Mini kit, QIAGEN GmbH, Hilden, Germany). The concentration of the extracted DNA was measured by Nanodrop (Labtech; UK) @260 and 280 nm. Bisulfite conversion of each DNA templates was performed using the Qiagen EpiTect Bisulfite Kit (Cat. 59104, Qiagen, Germany), according to the manufacturer’s instructions. The modified DNA was stored at −20°C until use.

**Methylation-Specific Polymerase Chain Reaction (MSP)**

The promoter hypermethylation of MALAT1 was detected by methylation-specific PCR (MSP) assay utilizing the above-mentioned bisulfite modified DNA as a template. We analyzed the MALAT1 sequence with UCSC genome browser, where the CpG islands 441bp was found at position chr11:65497487-65497927, while the CpG islands 441bp within the upstream of the human MALAT1 gene. MethPrimer (http://www.urogene.org/methprimer/) and Gene Runner software (version 4.0.6.68 Beta) were used to design two primer pairs, each specifically designed to attach to methylated or unmethylated strands. Our MSP designed primers were 5′- TTTCGGCGTTTGTTTTTGAC-3′ (forward) and 5′- AACTAAAACTTCCCGACGC-3′ (reverse) for the methylated sequence of MALAT1, while they were 5′- TGGTGTTTGTTTTTGATGTAG-3′ (forward) and 5′-AACTAAAACTTCCCAACACC-3′ (reverse) for its unmethylated sequence.

**Figure 1:**
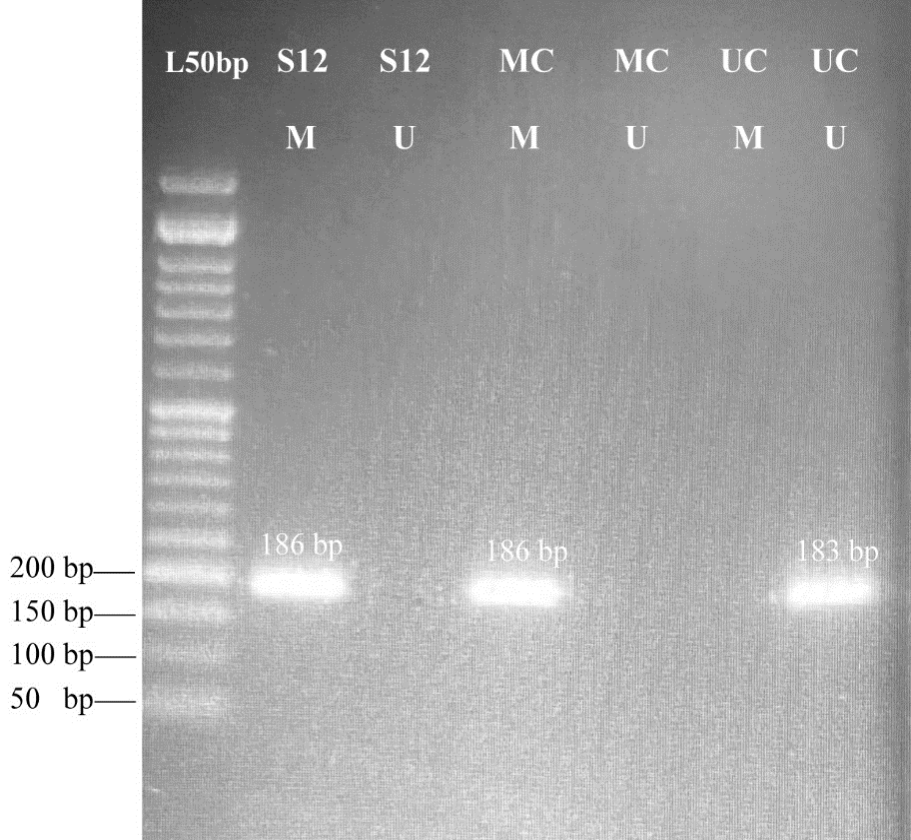
Agarose gel electrophoresis of methylation specific PCR products for MALAT1 methylated and unmethylated promoter; L, DNA ladder 50 bp; M, methylated primer; U, Un-methylated primer, MC, methylated control sequence; UC, unmethylated control sequence

Then, PCR amplification with MSP primers was performed using 11 μl of Master Mix and 1 .5μl of bisulfite-converted DNA in a final reaction mixture of 12.5 μL. The master mix included 0.5  μL of the forward primer (10 pmol concentration), 0.5 μl of the reverse primer (10 pmol concentration),1.25 μl of 10x MSP PCR buffer, 0.25 μL of 10  mM dNTP Mix, 0.5 μL of Mg, 2.5  μL of Q PCR buffer, 7.4 μl of nuclease free H2O, and 0.1μL of HotStart Taq DNA Polymerase (Qiagene, Germany). The optimal annealing temperature for both forward and reverse primers was 61ᵒC; the product size for the methylated primer was 186bp while that of unmethylated primer was 183bp. EpiTect PCR Control DNA Set (EpiTect PCR control DNA; Qiagen; Cat No; 59695) was utilized as a positive control for methylation and negative control for fully unmethylated DNA. PCR products were evaluated by electrophoresis on green viewer stained 2% agarose gel and visualized with a UV transilluminator (Figure 1). All of the samples were amplified twice for checking the accuracy of results. Occurrence of two bands in both M and U wells indicates Hemi methylated alleles which are supposed to be due to contamination with the normal colonic mucosa.

**Statistical Analysis**

Variables were reported as number and percentages. The extent of methylation of *MALAT1* in different tissue groups and clinicopathologic parameters were evaluated using the χ2 or Fisher exact test. P < 0.05 was considered as a statistically significant difference. Dataset was analyzed using SPSS (SPSS Inc. Chicago, Illinois, USA).

**Table 1:** Evaluation of *MALAT1*, promoter methylation pattern in tumor tissue and polyp tissue

Variable	*MALAT1*		
	Methylated, n (%)	Unmethylated, n (%)	P-value
Normal & Patient Tissues			0.430
Normal Tissue	4 (20.0)	16 (80)	
Primary Lesions	16 (34.8)	30 (65.2)	
Tumor tissue	5 (25)	15 (75)	
Polyp Type & Tumor tissues			0.437
Adenomatous polyp	9(39.1)	14(60.9)	
Hyperplastic polyp	4(30.8)	9(69.2)	
Inflammatory polyp	3(30)	7(70)	
Tumor tissue	11(55)	9(45)	

*MALAT1*, metastasis-associated lung adenocarcinoma transcript 1

**Table 2:** Correlation between clinicopathological characteristics and *MALAT1*, promoter methylation pattern in the 46 patients with colon polyps

Variable	*MALAT1*		P-value
	Methylated, n (%)	Unmethylated, n (%)	
Family history of colon polyps			0.143
Yes	6 (66.7)	3 (33.3)	
No	10 (38.5)	16 (61.5)	
Type of polyp			0.481
Adenomatous	11 (47.8)	12 (52.2)	
Hyperplastic	4 (30.8)	9 (69.2)	
Inflammatory	3 (30.0)	7(70.0)	
Location of polyp			0.035
Colon	14 (51.9)	13(48.1)	
Rectum	4 (21.1)	15 (78.9)	
Number of polyps			0.531
<5	12 (38.7)	19 (61.3)	
5-10	5 (50.0)	5 (50.0)	
>10	1 (20.0)	4 (80.0)	
Size of polyp			0.979
<5 mm	3 (37.5)	5 (62.5)	
5-10 mm	5(38.5)	8 (61.5)	
>10 mm	5 (41.7)	7 (58.3)	

*MALAT1*, metastasis-associated lung adenocarcinoma transcript 1

**Table 3:** Correlation between clinicopathological characteristics and *MALAT1*, promoter methylation pattern in 20 patients with colon cancer tissue

Variable	*MALAT1*		P-value
	Methylated, n (%)	Unmethylated, n (%)	
Family history of colon cancer			0.197
Yes	2 (50)	2 (50)	
No	3 (18.8)	13 (81.3)	
Tumor site			1
Colon	3 (25)	9 (75)	
Rectum	2 (25)	6 (75)	
Grade			0.436
I	3 (33.3)	6 (66.7)	
II & III	2 (22.2)	7 (77.8)	
Stage			0.795
I & II	3 (27.3)	8 (72.7)	
III	2 (22.2)	7 (77.8)	
Size of tumor			0.573
<5 cm	2 (33.3)	4 (66.7)	
≥5 cm	3(21.4)	11 (78.6)	

*MALAT1*, metastasis-associated lung adenocarcinoma transcript 1

## Results

**General Statistical Information**

This study was performed on 86 fresh/frozen colorectal tissue samples consisting of 46 polyps (24 (52.2%) male and 22 (47.8%) female), 20 tumours (7 (35%) male and 13 (65%) female), and 20 normal (8 (9.2%) male and 13 (14.9%) female). The polyp group consisted of 28.3, 50, and 21.7% hyperplastic polyps, adenomas and inflammatory polyp tissues, respectively.

The mean age of the patients with polyp and tumor was 49.71 (SD: 17.54) and 56.50 (SD: 15.61) years and the normal was 55.5 (SD: 11.13), respectively; their means (x̄) and SDs based on Body Mass Index (BMI) were 25.74±3.42 and 25.51±2.38 Kg/m2 and the normal was 25.6±4.29 Kg/m2, respectively. In our research, 9 (19.6%) of the polyp participants had a family history of cancer and among the 20 tumor samples, only a small number of CRC patients 4 (16%) indicated a family history of cancer. Colonoscopic findings of patients with colorectal polyps showed that patients had several polyps: 31 (67.4%) had less than 5 polyps, 10 (21.7%) had 5 to 10, and 5 (10.9%) had more than ten polyps. Furthermore, 27 (58.7%) of the primary lesions were located in colon region and 19 (41.3%) located in rectum. In the patients with colon cancer,12 (60%) had colon tumor and 8 (40%) had rectum tumor. Accurately, 6 (30 %) and 14 (70%) colon cancer individuals showed tumor size of <5 and **≥**5 cm, respectively. Among the colon cancer patients, 9 (45%) cases were classified as stage I, 2 (10%) stage II, and 9 (45%) stage III. The tumor grade was I, II, and III in 9 (45%), 9 (45%) and 2 (10%) cases, respectively.

**Analysis of MALAT1 promoter methylation status in normal compared to early colorectal lesions / tumor tissue**

The methylation frequency of *MALAT1* was 25% in cancerous tissues, 34.8% in primary lesion tissue, and 20% in normal tissues, respectively. The Chi-square test did not show any significant differences between cancerous tissues, primary lesion tissue, and normal tissue (p=0.430). In addition, polyp tissues categorized as pathological report to hyperplastic polyps, adenomas and inflammatory polyp tissues and methylation pattern of promotor *MALAT1* compared with tumor group, determination of methylation percentage in each group showed that, 39.1%, 30.8%, 30%, 55% of hyperplastic, adenomas, inflammatory polyp tissues and tumor tissues were methylated, respectively. However, none of these differences were statistically significant (p=0.437). Table 1 provides more details. 

**Evaluation of the relationship between the methylation of the MALAT1 promoter and polyp clinical characteristics**

According to our findings, *MALAT1* methylation was significantly higher in colon lesions in comparison with their rectum lesions, **p = 0.035**. The methylation frequency among colon and rectum region was 51.9% and 21.1%, respectively. Although 62.5 % and 61.5% of ≤ 5 mm and 5-10 mm polyp sizes were unmethylated, there was no significant difference between the size of the lesions. No significant correlations were observed either between the DNA methylation of *MALAT1* promoter and the other patients’ clinicopathological data, such as family history (FH), type of polyp, and number of polyps (Table 2). 

**Evaluation of the relationship between the methylation of the MALAT1 promoter and tumor spacemen’s clinical characteristics**

The specimens that exhibited invasion (20 tumor tissues) had an equal frequency of *MALAT1* unmethylation as compared to the group without family history of colon cancer, and this difference was not statistically significant (P = 0.197). Grade I tumors had a higher frequency of methylation, but no statistically significant difference was observed between the frequency of *MALAT1* unmethylation and grade I and II & III tumor tissue (P = 0.436). On the other hand, none of clinicopathological variables showed any correlation with *MALAT1* methylation status in the tumor tissue (Table 3).

## Discussion

LncRNAs play an important role in different types of cancers (27). MALATfig1 is one of the most important LncRNAs located on chromosome 11q13.1; it lacks an (open reading frame) ORF, thus making translation impossible (28). The main function of this gene is not completely known. However, this gene plays different roles in different cancers. In cervical cancer, it causes cell proliferation and invasion (29). MALAT1 promotes esophageal squamous cell proliferation and metastasis (19) (30) and can also increase glioblastoma cell migration (31). MALAT1 also increases migration and invades neuroblastoma cells (32). MALAT1 plays a critical role in the metastasis phenotype of lung, gastric, and bladder cancers (20, 33, 34). Recently, it has been observed that MALAT1 has a metastatic role in colorectal cancer (35). Numerous studies on this gene have shown that MALAT1 plays a vital role in tumor progression and can be used as a tumor prognostic marker. It has also been proved be overexpressed in most cancers. In a study, MALAT1 expression also significantly increased in lung cancer cells as compared to NC and adjacent lung cancer tissue (19). 

Therefore, research on methylation of this gene is important as gaining further knowledge and information can assist in developing diagnostic methods, as well as early detection and leads to the discovery of new epigenetic targeted drugs.

According to our findings, MALAT1 methylation was significantly higher in colon lesions in comparison with their rectum lesion. According to our knowledge, evaluation of aberrant *MALAT1* methylation pattern in different colon region of primary lesion has been done in our study for the first time. There have been many studies on the abnormal expression of *MALAT1* in a variety of diseases; however, studies on *MALAT1* promoter methylation and its role in cancer are scarce. Also, to the best of our knowledge, no studies have still focused on the association between the methylation in the promoter of *MALAT1* and the risk of colorectal cancer. 

According to a previous functional study by Liwen Hu et al., CpG island methylation occurs in the *MALAT1* promoter in esophageal cancer tissue which does not affect *MALAT1* expression, while amplification of *MALAT1* was reportedly significantly correlated with *MALAT1* over-expression (36). Furthermore, another study on lung cancer observed reduced *MALAT1* promoter methylation, which is subsequently associated with increased *MALAT1* expression (19). The reason for this inconsistency may be attributed to the cancer types. Recent evidence at the molecular level suggests that *MALAT1* lncRNA is recruited to regulate pre-mRNA splicing, though this outcome is not supported by *MALAT1* mice experimental model systems (37, 38).

Yang et al. reported that silencing *MALAT1* can inhibit colon cancer proliferation, growth, and metastasis (39). Also, Zheng et al. demonstrated that the expression of *MALAT1* in colon cancer tissues is higher in stage II&III and can be used as a biomarker of stage II & III prognosis (40).

According to the information obtained in our study, we examined the association between *MALAT1* gene promoter methylation pattern and clinicopathologic factors including family history of colon cancer, tumor site, size, grade and stage in 20 patients with colorectal cancer. There was no significant relationship between these parameters and *MALAT1* methylation pattern. We propose that other molecular and epigenetic mechanisms may be involved in *MALAT1* gene sequencing. 

A possible explanation for this might be associated with single nucleotide Polymorphism (SNP). Previous studies *have* *reported*, SNP in promoter region of functional lncRNAs that may have been associated with cancer susceptibility through impact on the stability and efficiency of transcription (41, 42). Li Y et al. investigated the impact of association between genetic variations in the MALAT1 promoter region and colon cancer risk. Li Y et al. have reported rs1194338 was significantly correlated to reduced susceptibility of colon cancer, with an odds ratio (OR) value of 0.70 [95% confidence interval (CI)=0.49-0.99] (43).

In addition, *MALAT1* lncRNA is subject to post-transcriptional 5-methylcytosine (m5C) modifications, but the functional reasons for these changes remain unknown (44). Most studies in the field of molecular mechanisms have reported that *MALAT1* regulates tumor progression and metastasis through the following: 1) *MALAT1* serves a competitive endogenous RNA that contains many putative binding sites of miRNAs; 2) *MALAT1* interacts with Polycomb repressive complex 2 (PRC2) which catalyzes histone H3K27 methylation, which plays important roles in transcriptional repression and cancer; 3) signaling pathways including PI3K-AKT, MAPK, WNT, and NF-κB have been reported to be regulated by *MALAT1* in cancer (45-47). 

Nevertheless, we suggest that another possible area of future research would be to investigate why a significantly higher methylation frequency occurred in colon lesions as compared with their rectum counterparts.
